# Association of Genetic Risk Variants in SETBP1, FANCM, and LSP1 with Familial Breast Cancer in the Pakistani Pashtun Population

**DOI:** 10.12669/pjms.41.9.12324

**Published:** 2025-09

**Authors:** Hussain Shah, Muhammad Saeed, Asif Jan, Zakiullah

**Affiliations:** 1 Hussain Shah Department of Pharmacy, University of Peshawar, 25000, Peshawar, Pakistan; 2 Muhammad Saeed Department of Pharmacy, Qurtuba University Peshawar, Pakistan; 3 Asif Jan Department of Pharmacy, District Head Quarter (DHQ) Hospital Charsadda, 24430, Pakistan. University of Peshawar, 25000, Peshawar, Pakistan; 4 Zakiullah Department of Pharmacy, University of Peshawar, Peshawar, Pakistan

**Keywords:** Breast cancer, SETBP1, FANCM, LSP1, whole exome sequencing, Pashtun, genes

## Abstract

**Background and Objective:**

Familial breast cancer is often linked to inherited gene mutations. These mutations significantly increase the risk of developing breast cancer within affected families. The objective of this study was to identify genetic risk variants in SETBP1, FANCM, and LSP1 genes and its association with familial breast cancer in Pakistani Pashtun population.

**Methods:**

This case-control study was conducted at the Genomic Center, Rehman Medical Institute, Peshawar, from January 10/2018 to February 01/2020. A total of n=172 individuals were included in the study, consisting of 86 breast cancer subjects (cases) and 86 Healthy participants (controls). Whole exome sequencing and follow-up association analysis was used to identify risk variants associated with breast cancer.

**Results:**

Exome sequencing identified four variants in genes of our interest (SETBP1, FANCM, and LSP1). Among these, the SNP rs11082414 in SETBP1 (crude P=0.011; adjusted p=0.003) showed a strong positive association with breast cancer in the Pakistani Pashtun population. Whereas SNPs rs34252356 in FANCM (crude P=0.260; adjusted p=0.199), rs793842 in LSP1 (crude P=0.220; adjusted p=0.173), and rs621679 in SETBP1 (crude P=0.112; adjusted p=0.207) showed no notable association with breast cancer in the study population.

**Conclusion:**

This research enhances our understanding of the genetic risk factors for breast cancer in the Pashtun population and underscores the potential importance of the SETBP1 rs11082414 variant in identifying individuals at risk. The identification of genetic biomarkers can facilitate the development of targeted drug therapies, improve early detection, and enhance the effective management of breast cancer.

## INTRODUCTION

The Breast cancer (BC) is an uncontrolled (malignant) growth of cells in the breast,^1^ and is the most common type of cancer and the second leading cause of death.^2^ BC is a major public health problem in the United States and other parts of the world. Currently, one in four deaths in the United States is due to BC^3^ and is the leading cause of cancer death among females in less developed countries.^4^ It accounts for more than 40,000 deaths each year. The incidence of breast cancer (BC) is generally highest in North America and Northern Europe, intermediate in Southern and Eastern Europe and South America, and lowest in Africa and Asia.^5^ In China, approximately one-fourth of all cancer-related deaths are attributed to breast cancer, with both incidence and mortality rates rising annually.^6^ A survey on BC mortality reported a 36.1% increase from 1970 to 2005.^6^ In India, breast cancer accounts for the highest mortality rate, estimated at 12.7 deaths per 100,000 women.^7^ Pakistan, being one of the most populous countries in the world, is also experiencing a surge in BC cases. It is estimated that one in nine women in Pakistan are diagnosed with breast cancer, primarily due to low socioeconomic status, unhealthy lifestyle choices, and reproductive health factors.^8^,^9^ Moreover, breast cancer accounts for 34.6% of all female cancers in Pakistani women.^10^

Several BC risk factors have been recognized for a long time.^11^ Age progression, poor life style and genetic factors are key determinants. A family background of BC augments a woman’s personal peril; Diagnosing at a younger age and a higher count of affected relatives intensifies her risk. Early menarche, delayed menopause, no history of childbirth and modestly postponed age at first birth, but persistently augment risk.^12^ Nursing, past benign breast condition and having dense tissue observed on a mammogram are connected to considerable elevations in BC.^13^ Alcohol, the sole dietary factor firmly established, also is linked with an increase risk for BC. Nevertheless, the most significant factor is family history (FH). Individual risk increases with increasing number of relatives affected with BC.^14^ The genetic factors contributing to breast cancer are well-studied in European populations, but they remain largely unexplored in the Pakistani population. This study is specially designed to evaluate the genetic predisposition between SETBP1 rs11082414, FANCM rs34252356 and LSP1 rs793842 and rs621679 and BC in the female population of Khyber Pakhtunkhwa, Pakistan. The Pashtun population was selected for this study because of its unique genetic makeup, additionally this population remains largely under-studied in genetic research, highlighting the importance of investigating potential disease-associated variants within this group.

## METHODS

A total of 172 indigenous Pakistani female subjects of Pashtun ethnicity (86 cases and 86 controls) were enrolled from various regions of Khyber Pakhtunkhwa, including Peshawar, Mardan, Charsadda, Bannu, Dir, Kohat, Swat, and Hangu. Cases were recruited from Institute of Radiotherapy and Nuclear Medicine (IRNUM), Hayatabad Medical Complex (HMC), and Khyber Teaching Hospital (KTH), while controls were selected from healthy volunteers attending free medical camps organized by Shaukat Khanum Memorial Hospital. Clinical and sociodemographic data-including age, age at menarche, menstrual duration, marital status, age at first pregnancy, and breastfeeding duration-were collected using a structured proforma.

### Inclusion & Exclusion criteria:

Female patients aged 25-80 years with histologically confirmed breast cancer and at least one first- or second-degree relative with the disease. Exclusion criteria included women without a family history of breast cancer or those younger than 25 or older than 80. Written informed consent was obtained from all participants

### Ethical Approval:

It was obtained from the. Ethical Committee of the Department of Pharmacy, University of Peshawar (Ref.# 503/EC-FLES-UOP/2022), and conducted in accordance with the Declaration of Helsinki.

Three (3) ml of whole blood was collected from all the participants following aseptic procedures. The collected blood was stored at -20 °C in a properly labeled EDTA tube.DNA was extracted using standard DNA extraction Kit (Novel Genomic DNA Mini kit-cat-No. NG-S250), following manufacturer protocol. Quality of DNA was checked using 1% Agarose gel (*BioRad, Germany*) and DNA quantity was evaluated using Qubit^®^ fluorometer using dsDNA high sensitivity kit (HS Qubit dsDNA Assay kit Cat. No. Q32851). The final concentration of DNA was standardized to 10 ng/μL.

DNA samples were pooled following previously described protocols.^15^,^16^ DNA from each participant, with an equimolar concentration of 100 ng, was pooled together. Pooling of DNA helps to minimize the cost, time of analysis and to simplify the process. The Illumina Nextera XT DNA Library Preparation kit was employed to prepare DNA libraries with paired ends (2×101 bp). (Cat-FC-131-1096, Cat-FC-142-1123) following the manufacturer’s protocol. Genomic DNA was fragmented by the transposome and cleaned up to remove transposome-adhered fragments, minimizing interference in subsequent steps. The DNA was subjected to 12 cycles of thermal PCR for amplification. Fragments smaller than 150-200 bp were separated out with paramagnetic beads during PCR. The DNA fragments of interest were captured using biotinylated probes, while non-specific fragments were eliminated. Quantification of the libraries was performed with an Agilent 2100 Bioanalyzer. The libraries were sequenced using an Illumina MiSeq-2000 platform, with data saved in FASTQ format.

The final variant calls were obtained from raw sequencing data using a custom-built in-house WES bioinformatics pipeline. CASAVA and Trimmomatic tools were used to process raw FASTQ files from the Illumina HiSeq, removing low-quality reads. Burrows-Wheeler Aligner (BWA) software - mem (v 0.7.13) was employed to align the filtered reads to the reference genome (hg19/GRCh37).^17^,^18^ The GATK Genotype tool was employed to call variants, which were then stored as a VCF file. The annotation of variants was performed using ANNOVAR on the variant calling file. An Illumina MiSeq-2000 platform was used for sequencing the libraries, and the results were saved as FASTQ files.

### Statistical Analysis:

IBM Statistical Package for Social Sciences version 24 was employed for the statistical analysis of data. The significant variables identified for analysis were age, period duration, age at menarch, marital status, age at 1^st^ pregnancy, number of childrens, address, duration of breast feeding and geographical area and variants in Familial breast Cancer gene. Numbers and percentages were used to describe categorical variable data. Hardy-Weinberg equilibrium (HWE) was assessed for all reported SNPs. The chi-square (χ²) test was applied to compare minor allele frequencies (MAFs) between cases and controls. SNPs and Breast cancer co-relation/association was determined using binary logistic regression. Statistically a probability of p < 0.05 was considered significant.

## RESULTS

The demographic and clinical characteristics of cases and controls are listed in Table-I and II. The mean ages of healthy subjects (controls) and breast cancer (BC) patients were 54.14 ± 10.91 and 56.02 ± 10.83 years, respectively. The highest incidence of BC, 40 cases (22.7%), was observed in patients aged 31-50 years. The second highest, 33 cases (18.8%), was in patients aged 14-30 years, while the lowest incidence, 19 cases (8.5%), was observed in patients aged 51 years and above (t-test p-value = 1.27). All study participants were female (86/86). The highest incidence of familial BC, 52 cases (29.5%), was seen in Group-I (Peshawar, Khyber Agency, FR Kohat, and Nowshehra), compared to 41 cases (23.3%) among controls. Group-III districts (Swat, Malakand, Hangu, and Bannu) had 18 cases (10.2%), while Group-II districts (Mardan, Swabi, and Charsadda) had the lowest incidence at 11 cases (6.3%) (t-test p-value = 0.08). Marital status also significantly affected BC incidence. Married females showed the highest incidence, with 69 cases (39.2%) (t-test p-value = 1.12), compared to 19 cases (10.8%) in unmarried females, as detailed in Table-I.

**Table-I:** Demographics information/details of study participants (cases and controls).

Variables	Groups	Control	Case	P-value
Age (years)	14-30 years	28 15.9%)	33(18.8%)	1.27
	31-50 years	41(23.3%)	40(22.7%)	
	51 and above years	19(10.8%)	15(8.5%)	
Maritalstatus	Married	71(40.3%)	69(39.2%)	1.12
	Single	17(9.7%)	19(10.8%)	
Address	Peshawar, Khyber Agency, FR Kohat and Nowshehra (Group-I)	41(23.3%)	52(29.5%)	0.08
	Mardan,Swabi and Charsadda (Group-II)	19(10.8%)	11(6.3%)	
	Swat, Malakand, Hango and Bannu (Group-III)	14(8.0%)	18(10.2%)	

Various clinico-pathological features, such as the duration of breastfeeding, number of children, age at menarche, and menstrual duration, were also studied (Table-II). The highest incidence of BC, 44 cases (25.0%) (p = 1.03), was seen in patients with a breastfeeding history of two years or more. This was followed by 31 cases (17.6%) in patients who did not breastfeed (single or married with no children) and 13 cases (7.4%) in patients with less than two years of breastfeeding. The number of children also influenced BC incidence; patients with 1-5 children had the highest incidence at 33 cases (18.8%) (p = 0.09), while those with no children (single or married but no child) had 31 cases (17.6%), and those with six or more children had 25 cases (14.2%). Age at menarche also affected BC incidence; females with menarche at ages 11-13 had the highest incidence, 57 cases (32.4%) (p = 1.99), while those with menarche at age 14 and above had a lower incidence, 31 cases (17.6%).

**Table-II:** Clinical Characteristics of the study participants (cases and controls).

Variables	Groups	Control	Case	P-value
Age at Menarche	11-13 years	59(33.5%)	57(32.4%)	1.99
	14 and above years	29(16.5%)	31(17.6%)	
Period Duration	28	60(34.1%)	70(39.8%)	0.06
	>28	28(15.9%)	18(10.2%)	
Age at 1^st^ Pregnancy	up to 18 years	51(29.0%)	55(31.3%)	1.01
	>18 years	37(21.0%)	33(18.8%)	
No Of Children	No Children (Single or Married but having no children)	26(14.8%)	30(17.0%)	0.09
	1-5 Children	34(19.3%)	33(18.8%)	
	6 and above Children	28(15.9%)	25(14.2%)	
Duration Of Breast Feeding	No feeding(Single, Married but having no children)	27(15.3%)	31(17.6%)	1.03
	less than 2 years	12(6.8%)	13(7.4%)	
	2 and above years	49(27.8%)	44(25.0%)	

Moving beyond the traditional genetic association studies and linkage analysis, we performed deep whole exome sequencing to investigate the genetic biomarkers associated with BC in Pashtun demographic of Khyber Pakhtunkhwa. The process of sequencing was completed with a read depth of 500X. Exome sequencing produces a substantial data which is difficult and laborious to process, hence the raw data was filtered out for easy downstream analysis. In the first step, exome sequencing identified gene list was filtered for gene of our interest/ selected genes (namely SETBP1, FANCM, and LSP1).

A total of n=4 variants were identified in SETBP1, FANCM, and LSP1. The variants identified includes rs11082414/ SETBP1 (G>G/C), rs7938342/ LSP1 (T>T/A), rs621679/ LSP1 (G>G/A) and one deletion in FANCM that is rs34252356 (TCTTA>TCTTA/T) detail documented in Table-III. The polymerase chain reaction-restriction fragment length polymorphism (PCR-RFLP) technique validated variants detected by WES. Following exome sequencing logistic regression model was used to find association of reported variants with BC. We documented positive association of SNP rs11082414/ SETBP1 (G>G/C) (Crude P = 0.01 and adjusted P = 0.03) with BC whereas SNP rs34252356/ FANCM (TCCTA>TCTTA/T) (Crude P = 0.26 and adjusted P = 0.19), rs7938342/ LSP1 (T>T/A) (Crude P = 0.22 and adjusted P = 0.17) and rs621679/ LSP1 (G>G/A) (Crude P = 0.11 and adjusted P = 0.20) showed no notable association with BC in this study population (Table-IV). Interestingly, when the combined effect of all three genes on genetic susceptibility to breast cancer (BC) was analyzed in the target population, it was found that the presence of FANCM and LSP1 increased the association of SETBP1 with BC by twofold. Details are provided in Table-V.

**Table-III:** Whole exome sequencing identified variants in selected genes (SETBP1, FANCM, and LSP1).

	Gene	Variant	Homo%	Hetero%	Variant Type	HGVSc	HGVSp	dbSNP ID
Cases	SET BP1	G>G/C	19.2	80.7	missense	c.691G>C	p.Val231Leu	rs11082414
Control	SET BP1	G>G/C	25	75	missense	c.691G>C	p.Val231Leu	rs11082414
Cases	LSP1	T>T/A	25	75	missense	c.102T>A	p.His34Gln	rs7938342
Control	LSP1	T>T/A	33.3	66.6	missense	c.102T>A	p.His34Gln	rs7938342
Cases	LSP1	G>G/A	20	80	missense	c.682G>A	p.Ala228Thr	rs621679
Control	LSP1	G>G/A	14.2	85.7	missense	c.682G>A	p.Ala228Thr	rs621679
Cases	FAN CM	TCTTA>TCTTA/T	0	100	splice_region_variant	c.4516-5_4516-2delCTTA	N/A	rs34252356
Control	FAN CM	TCTTA>TCTTA/T	33.3	66.6	splice_region_variant	c.4516-5_4516-2delCTTA	N/A	rs34252356

**Table-IV:** Association analysis/Logistic regression analysis (adjusted and un-adjusted) of the reported variants.

SNP/Gene	Genotype/Allele	Polymorphism	Cases(%)	Controls (%)	Unadjusted OR (95% CI)	P-value	Adjusted OR (95% CI)	P-value
rs11082414/SETBP1	No Mutation	Wild	42(23.9%)	64(36.4%)	Reference			
	G>G/C	Het	38(21.6%)	19(10.8%)	0.33 (0.17-0.64)	0.01	0.3 (0.15-0.6)	0.03
	G>C/C	Hom	8(4.5%)	5(2.8%)	0.41 (0.13-1.34)	0.14	0.41 (0.12-1.49)	0.17
rs34252356/FANCM	No Mutation	Wild	53(30.1%)	58(33.0%)	Reference			
	TCTTA>TCTTA/T	Het	33(18.8%)	25(14.2%)	0.69 (0.37-1.31)	0.26	0.65 (0.33-1.26)	0.19
	TCTTA>T/T	Hom	2(1.1%)	5(2.8%)	2.28(0.43-12.28)	0.33	2.72 (0.47-15.61)	0.26
rs7938342/ LSP1	No Muataion	Wild	32(18.2%)	42(23.9%)	Reference			
	T>T/A	Het	41(23.3%)	36(20.5%)	0.67 (0.35-1.27)	0.22	0.63 (0.32-1.23)	0.17
	T>A/A	Hom	15(8.5%)	10(5.7%)	0.51 (0.2-1.28)	0.15	0.49 (0.18-1.3)	0.15
rs621679/ LSP11	No Mutation	Wild	44(25.0%)	44(25.0%)	Reference			
	G>G/A	Het	33(18.8%)	39(22.2%)	0.71(0.25-1.91)	0.11	0.72 (0.29-2.23)	0.20
	G>A/A	Hom	11(6.3%)	5(2.8%)	0.61 (0.3-2.01)	0.25	0.28 (0.21-1.4)	0.13

**Table-V:** Combined effect of all three genes (SETBP1, FANCM and LSP1) on genetic susceptibility of BC.

Genotype/allele	Polymorphism	Case N (%)	Control N (%)	Unadjusted OR (95% CI)	P-value	Adjusted OR (95% CI)	P-value
SETBP1	Wild	42(23.9%)	64(36.4%)	Reference			
	Mutation	46(26.1%)	24(13.6%)	0.34 (0.18-0.64)	0.01	0.32 (0.16-0.61)	0.00
FANCM	Wild	53(30.1%)	58(33.0%)	Reference			
	Mutation	35(19.9%)	30(17.0%)	0.78 (0.42-1.45)	0.43	0.75 (0.39-1.42)	0.27
LSP1	Wild	32(18.2%)	42(23.9%)	Reference			
	Mutation	56(31.8%)	46(26.1%)	0.63 (0.34-1.14)	0.12	0.59 (0.32-1.11)	0.10
LSP11	Wild	44(25.0%)	44(25.0%)	Reference			
	Mutation	44(25.0%)	44(25.0%)	1 (0.55-1.81)	1.00	0.98 (0.52-1.82)	0.74

## DISCUSSION

In this case-control study, whole exome sequencing was used to identify genetic variants associated with breast cancer in the Pashtun population of Pakistan. A significant association was found between the single nucleotide polymorphism (SNP) rs11082414 (G>G/C) in the *SETBP1* gene and breast cancer (crude P = 0.01, adjusted P = 0.03), indicating its potential role as a genetic risk factor in this ethnic group. The SETBP1 gene, situated on the long arm of chromosome 18 at position 18q12.2, encodes the SET binding protein 1. Variations in this gene have been associated with a heightened risk of developing several medical conditions, including intellectual developmental disorders, Schinzel-Giedion syndrome, and breast cancer.^19^,^20^

Our findings are consistent with previous research by Uddin et al.,^21^ which also reported an association between *SETBP1* mutations and increased breast cancer risk. Similarly, international studies such as those by Piazza et al.^22^have identified *SETBP1* as a critical gene involved in oncogenic processes, although their focus was primarily on hematologic malignancies. However, there remains limited literature exploring the association between *SETBP1* and breast cancer specifically in South Asian populations, including Pakistan. This difference in available data may be attributed to population-specific genetic variations and a lack of genomic studies in underrepresented ethnic groups.

FANCM (Fanconi Anemia Complementation Group M) is a gene located on chromosome 14 at the position 14q21.2, spanning the regions 25-27. This gene encodes a protein that plays a crucial role in DNA repair mechanisms.^23^ FANCM mutations are connected to a heightened risk of breast cancer, particularly in cases of breast cancer.^24^,^25^ Additionally, FANCM mutations are associated with Fanconi Anemia (FA).^23^,^26^,^27^ In our study, we identified the single nucleotide polymorphism (SNP) rs34252356 in the FANCM gene, characterized by the variant (TCTTA>TCTTA/T) within our study population. However, a logistic regression analysis did not find a statistically significant association between this SNP and breast cancer in the target population (with crude P=0.26 and adjusted P=0.19).

LSP1 (Lymphocyte-Specific Protein 1) is a gene that is expressed in several immune cells, including macrophages, lymphocytes, endothelial cells, and neutrophils. This protein plays a key role in regulating the activation and movement (chemotaxis) of neutrophils, which are crucial for the body’s immune response. Previous studies have shown a strong correlation between variations in LSP1 and the incidence of breast cancer, suggesting its potential role as a genetic risk factor for this disease.^28^,^29^ In our study, we identified two specific mutations in the LSP1 gene: rs7938342 and rs621679. Upon examining the distribution of these minor alleles and their respective genotypes, we found no significant differences between breast cancer patients and the control group. Subsequent statistical analysis of these single nucleotide polymorphisms (SNPs) confirmed that neither mutation showed a significant association with breast cancer in our study population. This suggests that, within the context of our research, these LSP1 variants do not contribute to an increased risk of developing breast cancer.

### Strength of this study:

It is the first to report a significant association between SETBP1 rs11082414 and breast cancer risk in the Pashtun population, adding novel insights to the limited genomic data available for this under-studied ethnic group. Additionally, the use of whole exome sequencing enabled comprehensive detection of coding variants, increasing the depth and scientific relevance of the findings.

### Limitations:

Despite the valuable insights provided by this study on familial breast cancer among the Pashtun population, it has some limitations also that includes; relatively small sample size, the genetic associations identified in this study are specific to the Pashtun ethnic population of Pakistan. Similarly, the study focused on three genes (SETBP1, FANCM, and LSP1), some other breast cancer related genes are overlooked. Additional research with large sample size, and including study subjects from other Pakistani sub-population could provide a more comprehensive understanding of the genetic underpinnings of breast cancer.

## CONCLUSION

This study establish substantial link between SETBP1 rs11082414 and increase breast cancer in the Pashtun population of Pakistan. Our study will pave the path for future investigation and therapy of the breast cancer patients. In this analogy, the government should sketch a plan to screen and identify all the genetically susceptible individuals and launched proper campaign for the awareness of population through print and electronic media.
